# No evidence for the presence of genetic variants predisposing to psychotic disorders on the non-deleted 22q11.2 allele of VCFS patients

**DOI:** 10.1038/tp.2016.258

**Published:** 2017-02-21

**Authors:** M Guipponi, F Santoni, M Schneider, C Gehrig, X B Bustillo, W R Kates, B Morrow, M Armando, S Vicari, F Sloan-Béna, M Gagnebin, V Shashi, S R Hooper, S Eliez, S E Antonarakis

**Affiliations:** 1 Department of Genetic Medicine and Development, University of Geneva Medical School and University Hospitals of Geneva, Geneva, Switzerland; 2 Office Médico-Pédagogique Research Unit, Department of Psychiatry, University of Geneva Medical School, Geneva, Switzerland.; 3 Department of Psychiatry and Behavioral Sciences, State University of New York at Upstate Medical University, Syracuse, NY, USA; 4 Department of Genetics, Albert Einstein College of Medicine, Yeshiva University, Bronx, NY, USA; 5 Child Neuropsychiatry Unit, Department of Neuroscience, I.R.C.C.S Children Hospital Bambino Gesù, Rome, Italy; 6 Division of Medical Genetics, Department of Pediatrics, Duke University Medical Center, Durham, NC, USA; 7 Department of Allied Health, University of North Carolina School of Medecine, Chapel Hill, NC, USA; 8 Institut of Genetics and Genomics in Geneva (iGE3), Geneva, Switzerland

## Abstract

The velo-cardio-facial syndrome (VCFS) is caused by hemizygous deletions on chromosome 22q11.2. The VCFS phenotype is complex and characterized by frequent occurrence of neuropsychiatric symptoms with up to 25–30% of cases suffering from psychotic disorders compared with only ~1% in the general population (odds ratio≈20–25). This makes the 22q11.2 deletion one of the most prominent risk factors for schizophrenia. However, its penetrance for neuropsychiatric phenotypes is incomplete suggesting that additional risk factors are required for disease development. These additional risk factors could lie anywhere on the genome, but by reducing the normal diploid to a haploid state, the 22q11.2 deletion could result in the unmasking of otherwise recessive alleles or functional variants on the non-deleted 22q11.2 allele. To test this hypothesis, we captured and sequenced the whole 22q11.2 non-deleted region in 88 VCFS patients with (*n*=40) and without (*n*=48) psychotic disorders to identify genetic variation that could increase the risk for schizophrenia. Single nucleotide variants (SNVs), small insertions/deletions (indels) and copy number variants were called and their distributions were compared between the two diagnostic groups using variant-, gene- and region-based association tests. None of these tests resulted in statistical evidence for the existence of a genetic variation in the non-deleted allele that would increase schizophrenia risk in VCFS patients. Power analysis showed that our study was able to achieve >80% statistical power to detect association of a risk variant with an odd ratio of ⩾22. However, it is certainly under-powered to detect risk variant of smaller effect sizes. Our study did not provide evidence that genetic variants of very large effect size located on the non-deleted 22q1.2 allele in VCFS patients increase the risk for developing psychotic disorders. Variants with smaller effects may be located in the remaining 22q11.2 allele and elsewhere in the genome. Therefore, whole exome or even genome sequencing for larger sample size would appear to be the next logical steps in the search for the genetic modifiers of the 22q11.2-deletion neuropsychiatric phenotype.

## Introduction

The velo-cardio-facial syndrome (VCFS), also known as the 22q11.2-deletion syndrome (22q11.2DS), is the most common microdeletion syndrome affecting ~1 in 2000–4000 individuals.^1^ It is caused by hemizygous deletions on chromosome 22q11.2, which occur predominantly *de novo* (~90% of cases).^2^ The majority of individuals have a 3 Mb deletion, whereas others (~10%) have a nested 1.5 Mb deletion. The occurrences of 22q11.2 deletions are associated with non-allelic homologous recombination between specific segmental duplications.^3^

The VCFS phenotype is extremely diverse manifesting with variable expression and incomplete penetrance in multiple organs/systems such as velopharyngeal insufficiency with cleft palate, speech disorders, cardiac defects, microcephaly, short stature, typical facial appearance, auricular anomalies, cognitive problems and neuropsychiatric manifestations.^1^ Of interest, VCFS patients show a greatly increased risk of developing psychotic disorders, including schizophrenia (SCZ).^4^ In late adolescence and early adulthood, up to 30% of all individuals with VCFS develop psychotic disorders.^5^

SCZ is a severe mental disorder that causes abnormal cognition and perceptions and affect ~1% of the population worldwide.^6^ Although the exact etiology is largely unknown, its genetic component is likely to consist of a combination of common and rare risk variants of small or large effect size, respectively. The 22q11.2 deletion is known as one of the strongest genetic risk factors for SCZ.^7^ However, the exact mechanism by which this deletion increases the risk to develop psychotic disorders in some, but not all VCFS individuals is currently unknown.

Several variables, such as parental origin and size of the deletion as well as additional genomic variants could influence the neuropsychiatric outcome of the 22q11.2 deletion. Regarding the contribution of parental origin of the deletion, the results are mixed. Basset *et al.*^8^ found no evidence for an effect of parental origin of the 22q11.2 deletion on SCZ risk.^8^ However, Green *et al.*^9^ showed that maternal origin of the deletion was associated with an increased prevalence of psychotic disorders when compared with paternal origin.^9^

When considering the size of the 22q11.2 deletion, there appeared to be no relationship between the development of SCZ and the size of the deletion.^1^ Consistently, Green *et al.*^9^ showed that deletion size did not have an effect on the distribution of psychotic disorders between individuals with typical (3 Mb) versus atypical deletions.^9^

Finally, the presence of additional copy number variants (CNV) as potential genetic modifiers of the 22q11.2 neuropsychiatric phenotype has been studied in a sample of 100 VCFS patients (44 with SCZ).^8^ No enrichment of novel inherited CNV or evidence of *de novo* CNVs outside the 22q11.2 region was found indicating that hemizygosity of the 22q11.2 locus appears to be the major CNV-related risk factor for SCZ in VCFS individuals. In a similar study, Williams *et al.*^10^ performed genome-wide CNV analysis in a cohort of 48 VCFS cases (23 with psychosis) and showed that VCFS subjects with psychosis had a significant increase in the average size of CNV as well as an enrichment for CNV overlapping loci previously implicated in neuropsychiatric disorders.^10^

Another hypothesis, that has not yet been systematically investigated, lies in the fact that allelic variation, such as single nucleotide variants (SNVs), indels and CNVs, within the non-deleted 22q11.2 allele could influence the neuropsychiatric outcome of the 22q11.2 deletion. According to this hypothesis, individuals carrying recessive variants in genes and/or functional elements predisposing to psychotic disorders would become hemizygote for these variants in the presence of the 22q11.2 deletion.^11, 12^

To investigate the presence of genetic variants including SNVs, indels and CNVs that may contribute to the development of psychotic disorder in patients with VCFS, we have captured and sequenced the whole 22q11.2 non-deleted allele in 88 VCFS individuals, 40 who suffer from psychotic disorders and 48 who do not. Our study did not identify any genetic variants that would influence the variable penetrance of psychotic disorders associated with the 22q11.2 deletion suggesting that such variants may be located elsewhere in the genome.

## Materials and methods

### VCFS patient recruitment

VCFS individuals carrying the 3 Mb 22q11.2 deletion were recruited and psychiatrically assessed. This research project was approved by the ethics committee of all participating centers. All participants or legally authorized representatives provided their written informed consent.

Participants from the Geneva cohort were recruited in Switzerland, France, Belgium, and Great Britain through advertisements in patient associations or word of mouth. Participants from the Rome cohort were referred from the Genetic Clinical Unit of the Bambino Gesù Hospital and through advertisement in patient associations. Written informed consent from the participants and their parents was collected under protocols approved by local Institutional Ethical Review Boards. The Duke participants were recruited from the medical genetics clinics at Duke and neighboring medical centers in North Carolina as well as from the North Carolina 22q11 support group. All procedures and sample collection were performed after informed consent, under a protocol approved by the Duke Institutional Review Board. The SUNY participants were recruited from the International Center for the Evaluation, Treatment and Study of Velo-Cardio-Facial Syndrome at SUNY Upstate Medical School, as well as from 22q11DS support groups. Written informed consent from participants and their parents was collected according to approved protocols by the SUNY Upstate Institutional Review Board.

### 22q11.2 allele sequencing and variant calling

Genomic DNA extracted from blood was used. The entire repeat-masked sequence of the 3 Mb 22q11.2 deletion and flanking sequences (hg19 genomic coordinates: chr22: 18 400 394–22 600 038) was captured using a SureSelect custom design kit (Agilent Technologies, Santa Clara, CA, USA) (Supplementary bed file contains the chromosomal position of the captured regions after merging probes with overlapping coordinates). High-throughput sequencing was performed on a HiSeq2000 (Illumina, San Diego, CA, USA) using manufacturer's recommendations. Demultiplexed fastq files were obtained using the Illumina CASAVA v1.8.2 software and processed by our ‘in-house' pipeline running on the Vital-IT (http://www.vital-it.ch) Center for high-performance computing of the Swiss Institute of Bioinformatics (SIB). Specifically, Burrows–Wheeler Aligner (BWA) was used to align the sequencing reads to the human reference genome NCBI build (GRCh37/hg19). SAMtools was used to remove duplicate reads. SNVs and indels were called using bcftools and Pindel 0.2.4, respectively. The minimum number of independent reads required for variant calling was set at 4. SNV and indels were then collected in a unique VCF file and annotated using the ANNOVAR package.^13^

ERDS software was used to infer CNVs (http://www.utahresearch.org/mingfuzhu/erds/).^14^

### Association tests

All association tests performed were based on Fisher's exact test with Bonferroni correction for multiple testing set at ⩽5%.

## Results

### VCFS patients

VCFS individuals carrying the 3 Mb 22q11.2 deletion were included in the present study. Participants were separated in two extreme subgroups according to their clinical phenotype: control (*N*=48) versus cases (*N*=40). Minimal age of inclusion for the control group was set at 15 years to limit the presence of false negatives (that is, participants who will develop a psychotic disorder later in life). The participants' psychiatric status was assessed during a structured clinical interview with various clinical instruments, depending on the age and recruitment site: the Structured Clinical Interview for DSM-IV-TR Axis 1 Disorders (SCID-I) and/or the Diagnostic Interview for Children and Adolescent (DICA), along with the psychotic disorders supplement of the K-SADS-PL. Control participants were rated as having no or minimal lifetime history of any psychotic symptoms (hallucinations or delusions). On the other hand, cases met criteria for a DSM-IV psychotic disorder at the last available assessment. Clinical and epidemiological data are summarized in Supplementary Table 1.

### 22q11.2 chromosomal region-sequencing data

After the removal of duplicate reads, 20 (±8 s.d.) million mapped to the targeted chromosomal region. These reads represented an average coverage of at least 20 × for 98.2% (±1.75 s.d.) and 98.2% (±1.91 s.d.) of the hemizygote target region for VCFS_cases and VCFS_controls, respectively. On average, 4674 (min: 3578; max: 6568) and 4585 (min: 3375; max: 6381) high-quality variants (Samtools QS ⩾50 and Pindel QS ⩾600) were detected per individual in VCFS_cases and VCFS_controls, respectively. Supplementary Tables 2 and 3 summarize sequencing coverage and variant calling results.

### Power analysis

**Figure 1:**
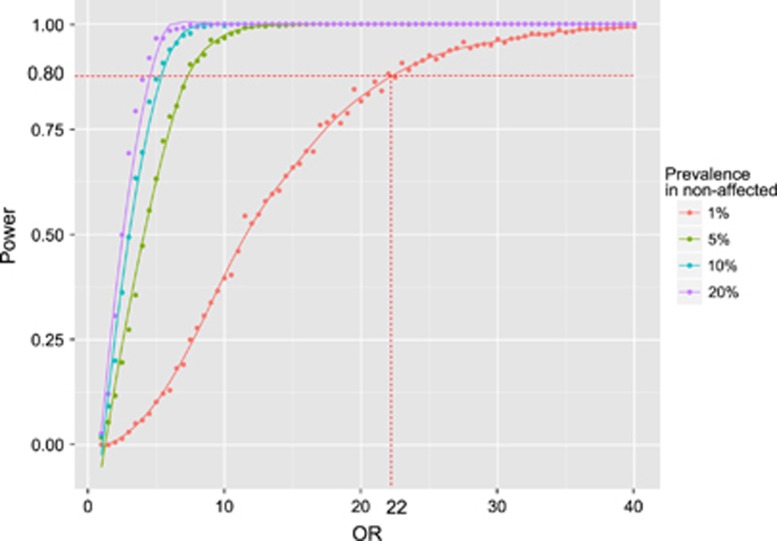
Power analysis. This study had 80% power to detect an odd ratio (OR) of 22.

The prevalence of psychotic disorder in the general population and in VCFS patients has been estimated as 1% and 30%, respectively,^4^ which corresponds to an odd ratio (OR) of ~30. Our sample size (40 cases and 48 controls) gives 80% statistical power to detect an OR>22 for a genetic variant with risk allele frequency of 0.01 at the 0.05 level of significance (simulation performed with power.fisher.test as implemented in the R software) Figure 1.

### Association analysis

#### Association tests for SNVs and indels

Given the prevalence of psychotic disorders in VCFS, only high-quality hemizygous variants with SAMtools and PINDEL quality scores of ⩾50 and ⩾600, respectively, and with a minimal allele frequency of ⩽30% in public databases (http://www.ncbi.nlm.nih.gov/SNP/ and http://exac.broadinstitute.org/) were retained for analysis. Moreover, variants present only once in our dataset (88 samples) were excluded. Only variants respecting the above-mentioned filters (a total of 4176) were considered in further analysis.

**Figure 2:**
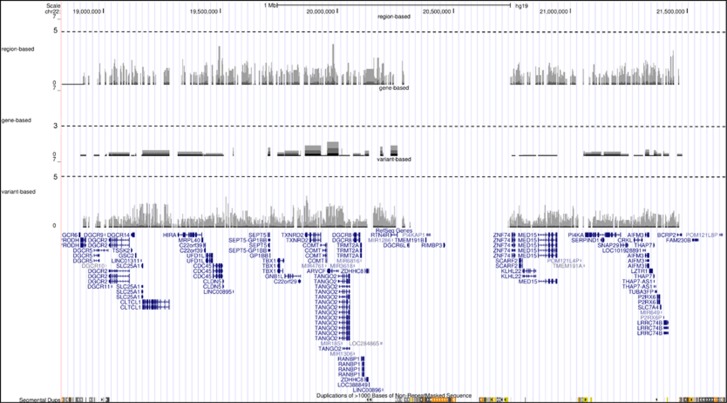
Log10 *P*-value plotted against chromosome 22q11.2 genomic coordinates (http://genome.ucsc.edu/) for the variant-, gene- region-based association test. Horizontal dotted lines show level of statistical significance corrected for the number of variants, genes and windows; *P*<10^−5^, *P*<10^−3^ and *P*<10^−5^, respectively.

Three different association tests based on Fisher's exact test were employed. (i) Variant-based association test that compares the frequency of each variant between cases and controls (significance threshold corrected for the number of variants: *P*<10^−5^), (ii) Gene-based association test that compares the burden of coding and splice-site variants (±6 nt from the donor-acceptor site) for each gene between cases and controls (significance threshold corrected for the number of genes (*n*=73) in the region 22q11.2: *P*<10^−3^) and (iii) Region-based association study that compares the burden of variant located within a sliding window of 1, 5 and 10 kb between cases and controls (significance threshold corrected for the number of windows (=number of variants): *P*<10^−5^). None of the three association studies performed (variant-, gene- and region-based) yielded a signal above the false discovery rate-corrected threshold of significance (Figure 2).

#### Association test for CNV data

Two different approaches were used to test for association of potential CNVs and psychotic disorders in VCFS patients. We first calculated for each sample the normalized coverage per nucleotide (number of reads overlapping the nucleotide/total number of reads) and applied the Fisher test to identify a statistically significant difference between VCFS_cases and VCFS_controls independently for each nucleotide. We then used ERDS to infer CNV in each sample. The coverage-based test did not identify any significant difference between cases and control. This result was confirmed by the analysis using ERDS in which neither deletions nor duplications were detected.

## Discussion

In this study, we sought to test the hypothesis that specific genetic variants, including SNVs, indels and CNVs, located on the hemizygous 22q11.2 region might modify the risk of VCFS individuals to develop psychotic disorder. We sequenced the entire hemizygous 22q11.2 allele in 88 subjects with the 22q11.2 deletion, 40 who suffer from psychotic disorders and 48 who do not. The distribution of genomic variants, including SNVs, indels and CNVs, were compared between the two diagnostic groups using several association tests. We did not observe any difference in the distribution of genetic variants in any of the association tests performed (variant-, gene-, region-based tests) between the two diagnostic groups. These results did not support the existence of specific genetic variants for the development of psychotic disorder in the non-deleted 22q11.2 allele in VCFS patients.

A limitation of our study resides in the fact that some of the VCFS_controls may not have yet entered the age when psychotic symptoms become more prevalent. The average age of onset for psychotic disorders in VCFS individuals varies between early adolescence and early adulthood with a mean in the late teens and early 20s.^15^ The mean age of the VCFS_controls at their last evaluation is 22.17±6.07 years and thus we cannot exclude that some might develop psychotic symptoms later in life, which may have deflated our power to detect a positive association signal.

We repeated this analysis by removing control individuals aged <20 years. The results were very similar to what obtained considering all individuals with no signal above the false discovery rate-corrected threshold of significance (data not shown). The two age distributions (mean±s.d.) were very similar: 22.8±6 years for the VCFS_cases versus 22.2±−6 years for the VCFS_controls.

On the basis of our statistical power calculation, our study appeared well-powered to detect association of the expected effect size. Indeed, the prevalence of psychotic disorder in the general population and in VCFS patients has been estimated as 1% and 30%, respectively.^4^ The odds of psychotic disorder are 30 × higher in VCFS patients than in the general population which corresponds to an OR of 30. Our sample size (40 cases and 48 controls) gives 80% power to detect one variant with an OR>22 at the 0.05 level of significance (Figure 2). However, our study design appeared definitely under-powered if the genetic architecture of the trait is made of many risk variants, each of small effect size.

Although not significant, the best association signals point to the *ARVCF* gene (chr22:19 '957 '402–20 '004 '309 (hg19); NM_001670, which is a member of the catenin family. This family of genes has an important role in the formation of adherens junction complexes, which are thought to facilitate communication between the inside and outside environments of a cell.^16^ Hence, ARVCF may have a role in intracellular signaling during embryonic development and be an integral part of the neurodevelopmental hypothesis for SCZ.^17^ Several studies have investigated the role of *ARVCF* in SCZ susceptibility. Sanders *et al.*^18^ suggest a possible role for ARVCF as a candidate gene for SCZ via alterations in neural development.^18^ Mas *et al.*^19, 20^ provided further evidence for an association of ARVCF with SCZ.^19, 20^ Interestingly, this gene has been recently shown to influence neurocognitive and neuroanatomical intermediate phenotypes in patients with SCZ.^21^ However, others studies did not find positive association between ARVCF and SCZ.^22, 23, 24^

Whether this finding represent genuine association signal or is simply an effect of an under-powered study arising from a modest sample sizes remains to be determined. Larger, sufficiently powered studies in independent cohorts would be needed to ascertain what role, if any, genetic variants in the hemizygous 22q11.2 allele have in the risk of VCFS patients for developing psychotic disorders.

Another line of research would be the study of epigenetic processes such as DNA methylation and histone modifications in brain tissues. Indeed aberrant epigenetic regulation and the resulting transcriptional dysregulation may contribute to the development of psychotic disorders in VCFS individuals. However, the investigation of these effects is beyond the scope of our study and presents non-trivial technical and ethical challenges related to the analysis of epigenetic signatures in brain tissues.

Our study suggests that the putative causative variant(s) of very large effect for SCZ risk in VCFS are located outside of the 22q11.2 hemizygote region. Variants with smaller effects may be located in the remaining 22q11.2 allele and elsewhere in the genome. Another possibility is that there may be neurocognitive and neuroanatomical intermediate phenotypes through which genetic variants with the non-deleted 22q11.2 region are directly or indirectly influencing risk for SCZ. In any case, exome or genome sequencing of large cohort of cases and controls may be considered.

## Supplementary Material

Supplementary Tables

Supplementary bed file
