# Supplementary material for: No evidence for the presence of genetic variants predisposing to psychotic disorders on the non-deleted 22q11.2 allele of VCFS patients
Source: Transl Psychiatry. 2017 Feb 21;7(2):e1039–. doi: 10.1038/tp.2016.258 (PMC5438018; doi:10.1038/tp.2016.258)
Supplement: Supplementary Tables [file tp2016258x1.docx]

**Supplementary Tables:**

**Supplementary Table 1: Clinical and epidemiological data**

| **Ind** | **Status** | **Gender** | **AOO** | **Clinical instrument used** | **Hallucinations/delusions** | **Psychotic disorder**  **(DSM-IV criteria)** | **Date last info** | **Age last info** |
| --- | --- | --- | --- | --- | --- | --- | --- | --- |
| VCFS_1 | Control | Male | n.a | K-SADS | Not present | None | Mar:2009 | 15 |
| VCFS_2 | Control | Male | n.a | K-SADS | Not present | None | Apr:2002 | 15 |
| VCFS_3 | Control | Female | n.a | SCID-1 | None | None | Aug:2015 | 18 |
| VCFS_4 | Control | Female | n.a | K-SADS | Not present | None | Nov:2005 | 16 |
| VCFS_5 | Control | Male | n.a | K-SADS_PL | None | None | Feb:2015 | 18 |
| VCFS_6 | Control | Female | n.a | SCID-1 | None | None | Aug:2015 | 20 |
| VCFS_7 | Control | Male | n.a | K-SADS | Not present | None | Feb:2016 | 20 |
| VCFS_8 | Control | Male | n.a | K-SADS_PL | None | None | Dec:2015 | 19 |
| VCFS_9 | Control | Female | n.a | K-SADS_PL | None | None | Dec:2015 | 19 |
| VCFS_10 | Control | Male | n.a | K-SADS_PL | None | None | Nov:2015 | 19 |
| VCFS_11 | Control | Female | n.a | SCID-I/K-SADS-PL | None | None | Oct:2011 | 18 |
| VCFS_12 | Control | Male | n.a | SCID-I/K-SADS-PL | None | None | Oct:2011 | 18 |
| VCFS_13 | Control | Male | n.a | SCID-I/K-SADS-PL | None | None | Jan:2010 | 19 |
| VCFS_14 | Control | Female | n.a | K-SADS_PL | None | None | Jun:2014 | 19 |
| VCFS_15 | Control | Male | n.a | K-SADS_PL | None | None | Feb:2014 | 19 |
| VCFS_16 | Control | Female | n.a | SCID-I/K-SADS-PL | None | None | Jun:2012 | 19 |
| VCFS_17 | Control | Female | n.a | SCID | Not present | None | Feb:2013 | 19 |
| VCFS_18 | Control | Male | n.a | SCID-I/K-SADS-PL | None | None | Aug:2011 | 19 |
| VCFS_19 | Control | Female | n.a | SCID-I/K-SADS-PL | None | None | Nov:2013 | 19 |
| VCFS_20 | Control | Male | n.a | SCID-I/K-SADS-PL | None | None | Jun:2012 | 20 |
| VCFS_21 | Control | Male | n.a | SCID-I/K-SADS-PL | None | None | Jun:2014 | 20 |
| VCFS_22 | Control | Male | n.a | K-SADS_PL | None | None | Sep:2014 | 20 |
| VCFS_23 | Control | Male | n.a | SCID | Not present | None | May:2015 | 23 |
| VCFS_24 | Control | Female | n.a | SCID | Not present | None | Jun:2013 | 21 |
| VCFS_25 | Control | Female | n.a | SCID | Not present | None | Apr:2013 | 21 |
| VCFS_26 | Control | Female | n.a | SCID | Not present | None | Mar:2013 | 21 |
| VCFS_27 | Control | Female | n.a | K-SADS_PL | None | None | Sep:2014 | 21 |
| VCFS_28 | Control | Female | n.a | SCID-I/K-SADS-PL | None | None | Nov:2013 | 21 |
| VCFS_29 | Control | Female | n.a | SCID-I/K-SADS-PL | None | None | Oct:2008 | 22 |
| VCFS_30 | Control | Male | n.a | K-SADS_PL | None | None | Mar:2013 | 22 |
| VCFS_31 | Control | Female | n.a | SCID-I/K-SADS-PL | None | None | Aug:2012 | 22 |
| VCFS_32 | Control | Female | n.a | SCID-I/K-SADS-PL | None | None | Jul:2013 | 23 |
| VCFS_33 | Control | Male | n.a | SCID-I/K-SADS-PL | None | None | Sept:2014 | 23 |
| VCFS_34 | Control | Male | n.a | SCID-I/K-SADS-PL | None | None | Aug:2013 | 23 |
| VCFS_35 | Control | Male | n.a | SCID-I/K-SADS-PL | Psychotic symptoms | None | Oct:2014 | 24 |
| VCFS_36 | Control | Female | n.a | SCID-I/K-SADS-PL | None | None | May:2013 | 24 |
| VCFS_37 | Control | Male | n.a | K-SADS_PL | None | None | Feb:2014 | 24 |
| VCFS_38 | Control | Male | n.a | SCID | Not present | None | Jan:2015 | 24 |
| VCFS_39 | Control | Female | n.a | SCID | Not present | None | Jan:2014 | 24 |
| VCFS_40 | Control | Male | n.a | SCID-I/K-SADS-PL | Subclinical Hallucination | None | Sept:2014 | 25 |
| VCFS_41 | Control | Male | n.a | SCID-I/K-SADS-PL | None | None | Jul:2014 | 25 |
| VCFS_42 | Control | Female | n.a | SCID | Not present | None | Oct:2012 | 28 |
| VCFS_43 | Control | Female | n.a | SCID | Not present | None | Jan:2003 | 31 |
| VCFS_44 | Control | Female | n.a | SCID | Subclinical Hallucination | None | Feb:2014 | 31 |
| VCFS_45 | Control | Female | n.a | SCID | Not present | None | Sep:2003 | 33 |
| VCFS_46 | Control | Male | n.a | SCID | Not present | None | Oct:2014 | 34 |
| VCFS_47 | Control | Female | n.a | SCID | Not present | None | May:2013 | 35 |
| VCFS_48 | Control | Female | n.a | SCID | Not present | None | Dec:2013 | 47 |
| VCFS_49 | Case | Female | n.a | SCID-1 | Psychotic symptoms | Psychosis | Dec:2006 | 16 |
| VCFS_50 | Case | Female | n.a. | K-SADS | Hallucinations and delusions | Psychotic disorder NOS | Dec:2013 | 17 |
| VCFS_51 | Case | Male | n.a | K-SADS | Delusions and subclinical hallucinations | Psychotic disorder NOS | Apr:2014 | 18 |
| VCFS_52 | Case | Male | n.a | SCID | Delusions and subclinical hallucinations | Schizophreniform disorder | Oct:2011 | 19 |
| VCFS_53 | Case | Female | 16 | K-SADS_PL | n.a | Psychosis | Mar:2015 | 19 |
| VCFS_54 | Case | Female | 15 | K-SADS_PL | n.a | Psychosis | Mar:2014 | 19 |
| VCFS_55 | Case | Female | 15 | K-SADS_PL | n.a | Psychosis | Feb:2014 | 20 |
| VCFS_56 | Case | Male | 15 | K-SADS_PL | n.a | Psychosis | Sep:2014 | 20 |
| VCFS_57 | Case | Female | n.a | SCID-I/K-SADS-PL | Hallucinations/Psychotic symptoms | Psychosis | Aug:2012 | 22 |
| VCFS_58 | Case | Female | n.a | SCID-I/K-SADS-PL | Hallucinations/Psychotic symptoms | Psychosis | Sep:2007 | 22 |
| VCFS_59 | Case | Male | n.a | SCID-I/K-SADS-PL | Hallucinations/Psychotic symptoms | Psychosis | Aug:2012 | 24 |
| VCFS_60 | Case | Female | n.a | SCID-I/K-SADS-PL | Hallucinations/Psychotic symptoms | Psychosis | Oct:2007 | 24 |
| VCFS_61 | Case | Male | 16 | K-SADS_PL | n.a | Psychosis | Feb:2014 | 25 |
| VCFS_62 | Case | Male | n.a | SCID-I/K-SADS-PL | Hallucinations | Psychosis | Oct:2007 | 26 |
| VCFS_63 | Case | Male | n.a | SCID-I/K-SADS-PL | Psychotic symptoms | Psychosis | Jul:2008 | 27 |
| VCFS_64 | Case | Male | 16 | K-SADS | Hallucinations and delusions | Schizophrenia | Jun:2008 | 16 |
| VCFS_65 | Case | Female | n.a | SCID-I/K-SADS-PL | Hallucinations/Psychotic symptoms | Schizophrenia | Sept:2009 | 20 |
| VCFS_66 | Case | Female | 19 | SCID | Hallucinations and delusions | Schizoaffective disorder | Mar:2013 | 21 |
| VCFS_67 | Case | Male | 13 | K-SADS | Hallucinations and delusions | Schizophrenia | Feb:2005 | 16 |
| VCFS_68 | Case | Male | n.a | SCID-1 | Psychotic symptoms | Schizophrenia | Feb:2012 | 17 |
| VCFS_69 | Case | Male | 15 | K-SADS_PL | n.a | Schizophrenia | Sep:2014 | 18 |
| VCFS_70 | Case | Male | n.a | SCID-1 | Hallucinations/Psychotic symptoms | Schizophrenia | Mar:2012 | 18 |
| VCFS_71 | Case | Male | n.a | SCID-I/K-SADS-PL | Hallucinations/Psychotic symptoms | Schizophrenia | Dec:2011 | 19 |
| VCFS_72 | Case | Female | n.a | SCID-I/K-SADS-PL | Hallucinations/Psychotic symptoms | Schizophrenia | Jul:2014 | 19 |
| VCFS_73 | Case | Male | n.a | SCID-I/K-SADS-PL | Hallucinations/Psychotic symptoms | Schizophrenia | May:2010 | 20 |
| VCFS_74 | Case | Male | 16 | SCID | Hallucinations and delusions | Schizophrenia | May:2013 | 20 |
| VCFS_75 | Case | Female | 15 | K-SADS_PL | n.a | Schizophrenia | Sep:2014 | 20 |
| VCFS_76 | Case | Female | 19 | K-SADS_PL | n.a | Schizophrenia | Sep:2014 | 23 |
| VCFS_77 | Case | Male | 17 | SCID | Hallucinations and delusions | Schizophrenia | Jan:2005 | 24 |
| VCFS_78 | Case | Female | 19 | K-SADS_PL | n.a | Schizophrenia | Mar:2014 | 24 |
| VCFS_79 | Case | Male | 24 | SCID | Hallucinations and delusions | Schizophrenia | Jan:2014 | 25 |
| VCFS_80 | Case | Female | 23 | SCID | Hallucinations and delusions | Schizophrenia | Apr:2014 | 27 |
| VCFS_81 | Case | Male | 24 | K-SADS_PL | n.a | Schizophrenia | Feb:2014 | 28 |
| VCFS_82 | Case | Male | 21 | SCID | Hallucinations and delusions | Schizophrenia | Nov:2014 | 28 |
| VCFS_83 | Case | Female | 18 | SCID | Hallucinations and delusions | Schizophrenia | Oct:2006 | 36 |
| VCFS_84 | Case | Male | n.a | SCID | Hallucinations and delusions | Schizophrenia | Mar:2005 | 37 |
| VCFS_85 | Case | Male | 18 | SCID | Hallucinations and delusions | Schizophrenia | May:2011 | 39 |
| VCFS_86 | Case | Female | 15 | K-SADS_PL | n.a | Schizoaffective | Sep:2014 | 19 |
| VCFS_87 | Case | Male | n.a | SCID-I/K-SADS-PL | Hallucinations/Psychotic symptoms | Schizoaffective | Nov:2007 | 21 |
| VCFS_88 | Case | Female | n.a | SCID | Delurium/Hallucinations/Psychotic symptoms | Schizoaffective disorder | Jun:2014 | 43 |

**Supplementary Table 2:**

Coverage statistics for the targeted chromosomal region (chr22: 18’400’394-22’600’038; hg19)

% covered 20x or 50x: Percentage of the targeted region covered at least 20x or 50x.

|  | **N=** | **Average Coverage** | | | **% covered 20x** | | | **% covered 50x** | | |
| --- | --- | --- | --- | --- | --- | --- | --- | --- | --- | --- |
|  |  | Min | Mean | Max | Min | Mean | Max | Min | Mean | Max |
| **VCFS_cases** | 42 | 138 | 643.3 | 1497 | 85 | 98.2 | 99.7 | 64.6 | 95.0 | 99.4 |
| **VCFS_controls** | 48 | 137 | 720.3 | 1530 | 85.6 | 98.2 | 99.7 | 64.5 | 95.2 | 99.4 |

**Supplementary Table 3:**

Variant classification (Samtools SNV QS>=50, PINDEL INDEL QS >=600 hemizygous on chr22)

| **Type of variant** | **VCFS_cases** | | | **VCFS_controls** | | |
| --- | --- | --- | --- | --- | --- | --- |
|  | **Min** | **Mean** | **Max** | **Min** | **Mean** | **Max** |
| **Synonymous SNV** | 15 | 28.8 | 39 | 18 | 27.2 | 39 |
| **Non synonymous SNV** | 14 | 22.7 | 34 | 12 | 23.1 | 36 |
| **Stopgain** | 0 | 0.4 | 1 | 0 | 0.65 | 2 |
| **Stoploss** | 0 | 0 | 0 | 0 | 0 | 0 |
| **Frameshift insertion** | 0 | 0.1 | 1 | 0 | 0.2 | 2 |
| **Frameshift deletion** | 0 | 0.4 | 1 | 0 | 0.6 | 1 |
| **Frameshift substitution** | 0 | 0 | 0 | 0 | 0 | 0 |
| **Nonframeshift insertion** | 0 | 0.5 | 2 | 0 | 0.6 | 2 |
| **Nonframeshift deletion** | 0 | 0 | 0 | 0 | 0.04 | 1 |
| **Nonframeshift subst** | 0 | 0 | 0 | 0 | 0 | 0 |
| **Splicing (+/-5nt)** | 2 | 3.6 | 7 | 1 | 4.22 | 7 |
| **Intronic** | 1226 | 1643.6 | 2544 | 1034 | 1635.5 | 2450 |
| **All** | 3578 | 4674 | 6568 | 3375 | 4585 | 6381 |
